# PLK1 targets NOTCH1 during DNA damage and mitotic progression

**DOI:** 10.1074/jbc.RA119.009881

**Published:** 2019-10-09

**Authors:** Carlo De Blasio, Azzurra Zonfrilli, Matteo Franchitto, Germano Mariano, Samantha Cialfi, Nagendra Verma, Saula Checquolo, Diana Bellavia, Rocco Palermo, Dario Benelli, Isabella Screpanti, Claudio Talora

**Affiliations:** ‡ Department of Molecular Medicine, Sapienza University of Rome, Viale Regina Elena 291, 00161 Rome, Italy; § Department of Medico-Surgical Sciences and Biotechnology, Sapienza University, 04100 Latina, Italy; ¶ Center of Life Nano Science Sapienza, Istituto Italiano di Tecnologia, 00161 Rome, Italy

**Keywords:** Notch pathway, DNA damage response, cell cycle, cancer biology, stress response, Arsenic, cell transformation, DNA damage, Notch, PLK1

## Abstract

Notch signaling plays a complex role in carcinogenesis, and its signaling pathway has both tumor suppressor and oncogenic components. To identify regulators that might control this dual activity of NOTCH1, we screened a chemical library targeting kinases and identified Polo-like kinase 1 (PLK1) as one of the kinases involved in arsenite-induced NOTCH1 down-modulation. As PLK1 activity drives mitotic entry but also is inhibited after DNA damage, we investigated the PLK1-NOTCH1 interplay in the G_2_ phase of the cell cycle and in response to DNA damage. Here, we found that PLK1 regulates NOTCH1 expression at G_2_/M transition. However, when cells in G_2_ phase are challenged with DNA damage, PLK1 is inhibited to prevent entry into mitosis. Interestingly, we found that the interaction between NOTCH1 and PLK1 is functionally important during the DNA damage response, as we found that whereas PLK1 activity is inhibited, NOTCH1 expression is maintained during DNA damage response. During genotoxic stress, cellular transformation requires that promitotic activity must override DNA damage checkpoint signaling to drive proliferation. Interestingly, we found that arsenite-induced genotoxic stress causes a PLK1-dependent signaling response that antagonizes the involvement of NOTCH1 in the DNA damage checkpoint. Taken together, our data provide evidence that Notch signaling is altered but not abolished in SCC cells. Thus, it is also important to recognize that Notch plasticity might be modulated and could represent a key determinant to switch on/off either the oncogenic or tumor suppressor function of Notch signaling in a single type of tumor.

## Introduction

NOTCH signaling is essential for development, and it is a type of cell-cell signaling that participates in a wide range of biological processes from neurodegeneration to tumorigenesis (1, 2). The canonical *NOTCH* pathway is mediated by the regulated intramembrane proteolysis pathway, in which NOTCH receptors undergo ligand-dependent sequential endoproteolysis via different enzymes, including presenilin (PS)^4^/γ-secretase (3). The NOTCH-1 intracellular domain (ICD), which is produced by PS/γ-secretase–mediated cleavage at site 3 within the transmembrane domain, translocates to the nucleus to activate transcription of target genes (1, 2). Alteration of *NOTCH* signaling has been described as a major player in several human cancers (4). Furthermore, multiple lines of evidence indicate that *NOTCH* signaling is not exclusively oncogenic but can act as a tumor suppressor. In animal models, evidence for *NOTCH* signaling in mediating each of these roles has been established. Additionally, the NOTCH1 tumor suppressor role is also underlined by the loss or inactivating mutations of members of the *NOTCH* signaling pathway in human cancers, particularly in head and neck squamous cell carcinoma (HNSCC), in which inactivating mutations of *NOTCH1* were found in 10–15% of the tumors (5–10). Interestingly, a subset of HNSCC tumors with the *NOTCH1* WT sequence exhibit a *NOTCH* pathway copy number increase with activation of the downstream NOTCH targets, *HES1/HEY1* (5, 10). Additionally, inhibition of *NOTCH1* or *HEY1* significantly decreased cell growth of primary tumor-derived cells, indicating their potential involvement in HNSCC development (5, 10, 11). The molecular regulation of the dichotomous function of *NOTCH* signaling remains poorly understood. For this reason, we studied this dual activity of *NOTCH1* in arsenic-induced keratinocyte transformation, thus providing a model to investigate the molecular aspects determining whether *NOTCH* signaling will be either oncogenic or tumor-suppressive (12). We observed that the mechanism is characterized by two phases. The first phase involves the down-modulation of NOTCH1 expression, and the second phase involves the acquisition of resistance to arsenite-induced down-regulation of NOTCH1 (12). We found that maintenance of NOTCH1 expression supports metabolic activities to enhance cytoprotection against oxidative stress that as a side effect may sustain cell proliferation and keratinocyte transformation, strengthening the hypothesis that tumor cell selection could favor partial rather than complete inactivation of this signaling pathway (12). To identify regulators that may influence the dichotomous *NOTCH1* function, we screened a chemical library targeting human kinases and identified Polo-like kinase 1 (PLK1) as one of the kinases involved in arsenite-induced down-modulation of NOTCH1 expression. The Polo-like kinase is an important regulator of cell division responsible for a wide number of functions: centrosome maturation, DNA replication, mitotic entry, and adaptation to persistent DNA damage (13, 14). We identified NOTCH1 as a novel direct target of PLK1 kinase activity. *PLK1* inhibition reduced arsenite-induced NOTCH1 down-modulation. Arsenic is known to have genotoxic and mutagenic effects; genotoxic stress causes proliferating cells to activate the DNA damage checkpoint to assist DNA damage recovery by slowing cell cycle progression. Thus, to drive proliferation and transformation, cells must tolerate DNA damage and suppress the checkpoint response (see Ref. 15) and references therein). We report here that PLK1 promotes NOTCH1 down-modulation to the G_2_-M transition; conversely, NOTCH1 remains active during a DNA damage–induced G_2_ arrest. Our data show that NOTCH1 has pleiotropic effects in DNA damage-arrested cells, and also in those contexts where *NOTCH1* is known to play a tumor suppressor function, cancer cells might still be dependent on specific NOTCH1 signals to sustain their cancerous phenotype.

## Results

### PLK1 as a central kinase involved in arsenite-induced NOTCH1 down-modulation

**Figure 1.**
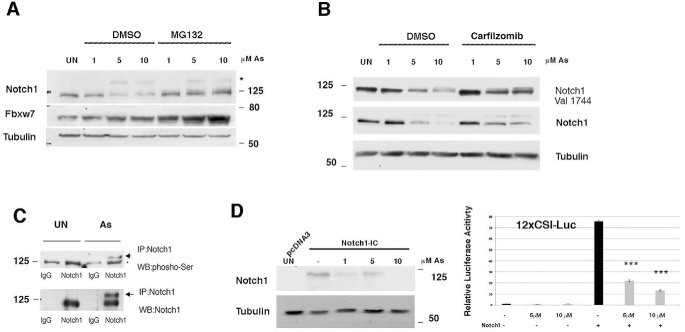
**Decreased NOTCH1 levels in As_2_O_3_-treated keratinocytes.** *A* and *B*, HaCaT cells were untreated or treated with As_2_O_3_ (*As*). 24 h post-treatment, cells were either untreated or treated with MG132/carfizomib for 5 h before collection; immunoblotting was performed with the indicated antibodies. *C*, HaCaT cells were treated with As_2_O_3_ for 24 h before collection, cell extract was immunoprecipitated (*IP*) using an antibody against NOTCH1, and immunoblotting (*WB*) was performed with the indicated antibodies. *D*, HaCaT cells were transfected with either pCDNA3 or NOTCH1-IC (encoding the human Notch1-IC, 1757–2555). 36 h post-transfection, cells were treated with As_2_O_3_ for 24 h before collection; immunoblotting was performed with the indicated antibodies. *D* (*right*), HaCaT cells were co-transfected with the *NOTCH*-responsive promoter 12xCSL, and the NOTCH1-IC plasmid was then treated with increasing amounts of As_2_O_3_ (5 and 10 μm) 12 h before collection. Average and S.D. values were calculated from triplicate samples. ***, *p* < 0.0001.

**Figure 2.**
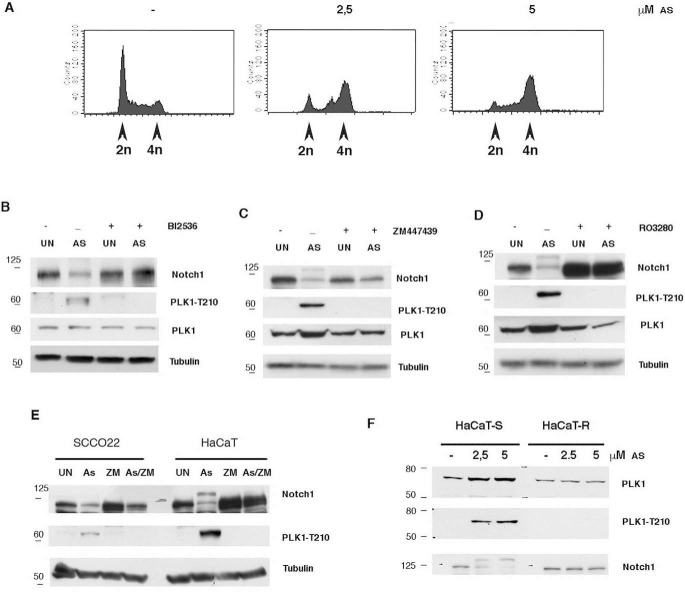
**Effects of PLK1 inhibition in As_2_O_3_-treated cells.** *A*, HaCaT cells were treated for 24 h with the indicated amount of arsenite, and then cells were collected, and the cell cycle was analyzed by FACS. *B–D*, immortalized HaCaT cells were treated with the indicated amount of As_2_O_3_ (*AS*) for 24 h, and then cells were treated with/without the indicated inhibitors (PLK1 inhibitor BI2536; ZM447439 Aurora A/B; RO3280 PLK1) for 24 h and analyzed by immunoblotting with the indicated antibodies. *E*, the indicated cell lines were treated with As_2_O_3_ for 24 h, and then cells were treated with/without 10 μm ZM447439 (*ZM*) for 24 h and analyzed by immunoblotting with the indicated antibodies. *F*, immortalized (HaCaT-S) and As_2_O_3_-transformed HaCaT cells (HaCaT-R) were treated with increasing amounts of As_2_O_3_ for 24 h and analyzed by immunoblotting with the indicated antibodies. Shown are representative results from at least three independent experiments. UN, untreated.

To explore the mechanisms that determine whether NOTCH signaling will be either oncogenic or tumor-suppressive, we used a well-defined *in vitro* model in which the nontumorigenic human keratinocyte cell line (HaCaT) was acutely exposed to arsenic trioxide (arsenite). We previously demonstrated that loss of FBXW7 induction might contribute to acquire both resistance to arsenite-induced down-modulation of NOTCH1 and HaCaT transformation (12). Here we show that arsenite stimulates the serine phosphorylation of NOTCH1 with the parallel decreased expression of NOTCH1 and up-regulation of FBXW7 levels (Fig. 1, *A–C*). Treatment of cells with the proteasome inhibitors prevented the decrease of NOTCH1 expression (Fig. 1, *A* and *B*). FBXW7 is a constituent of the SCF ubiquitin ligase complex (SKP1-CUL1-F box) that controls the degradation of NOTCH1. Substrate phosphorylation is required for FBXW7-mediated recognition (16–18). Thus, we developed a luciferase assay to identify the kinase that would prime NOTCH1 for recognition by FBXW7. First, HaCaT cells were transiently transfected with an expression vector of NOTCH1-IC. At 36 h after transfection, the cells were treated with arsenite for the last 12 h at the indicated concentrations (1, 5, and 10 μm). Total cell lysates were collected and subjected to Western blot analysis. Arsenite treatment decreased the NOTCH1 level compared with the vehicle-treated control cells (Fig. 1*D*), indicating that exogenous NOTCH1-IC is degraded similarly to the endogenous NOTCH1. Then we used a 12xCSL-luciferase reporter vector responsive to *NOTCH1* signaling, and we found that NOTCH1 transcriptional activity was strongly suppressed by arsenite treatment (Fig. 1*D*, *right*). This functional assay was used to screen a kinase inhibitor library of 378 small-molecule compounds. All compounds were screened in triplicate at 10 μm in the presence of 5 μm arsenite (data not shown). Those compounds showing at least a >50% recovery of luciferase activity were further tested by luciferase assay and Western blotting (Figs. S1 and S2). We identified 27 kinases able to rescue the NOTCH1 luciferase activity (Fig. S1). To understand the functional context of how the identified kinases might have an impact on NOTCH1, we performed a network analysis in which we investigated all possible direct and indirect interactions among them. For this purpose, the full Pathway Commons database of reported protein interactions in Simple Interaction Format (SIF) was performed. This analysis resulted in a network comprising 611 proteins with 2263 interactions (Fig. S3). The central component of the shortest path network was the protein PLK1. PLK1 is a promitotic kinase, and its main function is to facilitate the mitotic process (13, 14). However, PLK1 also promotes cell cycle progression in cells under stress conditions, thus facilitating tolerance to genotoxic stress (15). Arsenic is known to have genotoxic and mutagenic effects, and we observed that arsenite-treated cells were arrested in G_2_ (12) (Fig. 2*A*). Thus, we tested whether PLK1 activity might affect NOTCH1 expression following arsenite treatment. PLK1 activation requires phosphorylation on a conserved threonine in the T-loop of the kinase domain (Thr-210). PLK1 is first phosphorylated on Thr-210 in G_2_ phase by the kinase Aurora-A, in concert with its cofactor Bora (19, 20). Thus, to further characterize the pattern of Thr-210 phosphorylation and NOTCH1 stability, HaCaT cells were treated with arsenite and cultured in the presence or absence of both PLK1 and Aurora inhibitors. In agreement with the luciferase assay, accumulation of NOTCH1 protein upon treatment with PLK1 inhibitors was observed in arsenite-untreated and -treated HaCaT cultures as well as in SCC022, a squamous cell carcinoma–derived cell line (Fig. 2, *B*, *D*, and *E*). We previously demonstrated that arsenite-transformed keratinocytes acquire resistance to arsenite-induced NOTCH1 down modulation. Here, we observed PLK1 activation and NOTCH1 down-regulation after arsenite treatment in the presence of DNA damage signals, as shown by increased γ-H2AX (Fig. S5). We also found that PLK1 activation was not observed in arsenite-transformed keratinocytes (HaCaT-R) after arsenite treatment (Fig. 2*F*). This indicates that PLK1 activity might make a potential contribution at the early stages of arsenite carcinogenesis and that in arsenite-transformed keratinocytes PLK1 is no longer required in response to arsenite treatment, as cells have acquired a molecular switch required for cellular adaptation to genotoxic stress (*e.g.* metabolic adaptation) (12).

### NOTCH1 is a direct target of PLK1

Analysis of the NOTCH1 C-terminal primary amino acid sequence by different computational platforms revealed the presence of multiple potential phosphorylation sites for the PLK1 consensus sequences (R*XX*(pS/pT)*X*R*XX*R). However, to narrow down the number of candidate motifs prior to experimental verification, we analyzed the NOTCH1 protein sequence by considering as putative candidate motifs only those identified via a high-stringency analysis and that can be recognized by both the PhoshoNET and GPS-Polo 1.0 platforms. Two sites, Ser-1791 and Ser-2349, were identified by these criteria (Fig. S4, *A–C*). Interestingly, both motifs are conserved across species, and Ser-1791 was found to be phosphorylated also in colon cancer cells (21). To confirm that NOTCH1 can be phosphorylated by PLK1, we performed an *in vitro* kinase assay using purified recombinant PLK1 and NOTCH1-IC fragment as substrate. As shown in Fig. S4*D*, the C-terminal NOTCH1 fragment was readily phosphorylated by PLK1. Additionally, when the two putative phosphorylation sites, Ser-1791 and Ser-2349, were replaced by Ala, WT NOTCH1-IC but not the mutant was efficiently phosphorylated (Fig. S4*E*).

To test whether the phosphorylation of NOTCH1-IC on the putative PLK1 phosphorylation sites determined the stability of NOTCH1-ICD cells expressing either WT NOTCH1-IC or mutants, NOTCH1-IC-A1791/A2349 constructs were treated with cycloheximide. At various time points thereafter, the transfected cells were lysed, and the amounts of the NOTCH1 proteins were measured by Western blot analysis. We found that mutation of Ser-1791/2349 promotes NOTCH1-IC stabilization (Fig. S4*F*).

### NOTCH1 is a substrate of PLK1 in the G_2_ phase of the cell cycle

**Figure 3.**
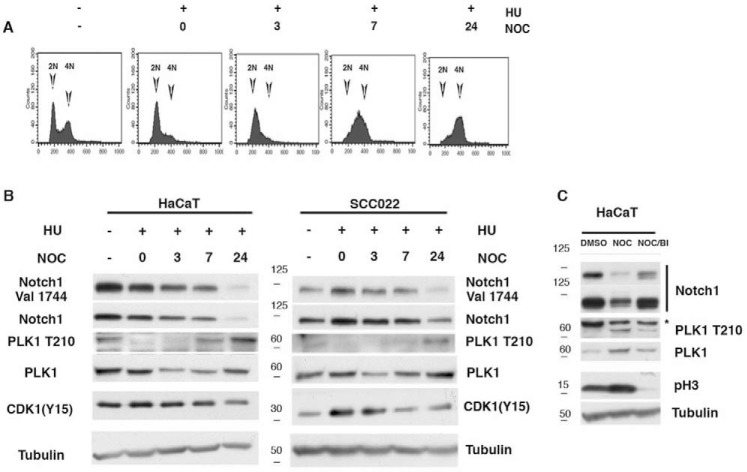
**PLK1-dependent degradation of NOTCH1 at the G_2_-M transition.** *A* and *B*, HaCaT and SCCO22 cells were collected at the indicated time points after release from G_1_/S, cell cycle was analyzed by FACS (FACS profile is shown only for HaCaT cells), and cell lysates were immunoblotted with antibodies to the indicated proteins. *C*, HaCaT cells were treated for 16 h with nocodazole to induce a mitotic block, and BI2536 (PLK1 inhibitor) was added 8 h before harvesting. Prometaphase cells were then collected by shake-off, and cell extracts were analyzed by immunoblotting with antibodies to the indicated proteins. Shown are representative results from at least three independent experiments. *NOC*, nocodazole; HU, hydroxyurea.

**Figure 4.**
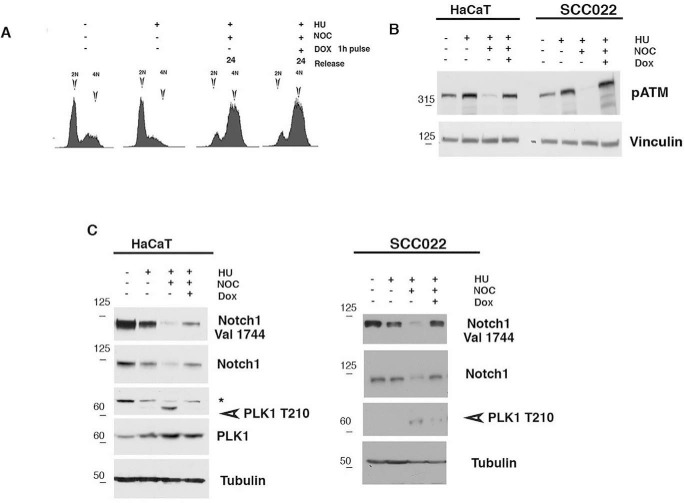
**NOTCH1 expression in G_2_ DNA damage arrest.** *A–C*, HaCaT cells were left untreated (diagram 1) or treated with hydroxyurea (*HU*) for 19 h (*A*). Alternatively, cells were released from the hydroxyurea block and either untreated or treated after 7 h with doxorubicin (*DOX*) for 1 h and subsequently grown in the presence of nocodazole (*NOC*) for 18 h. Following these treatments, cells were collected at the indicated time points after release from G_1_/S, cell cycle was analyzed by FACS (FACS profile is shown only for HaCaT cells), and cell lysates were immunoblotted with antibodies against the indicated proteins (*B* and *C*). Shown are representative results from at least three independent experiments.

To understand the functional significance of PLK1-mediated regulation of NOTCH1, we focused our attention to the PLK1/NOTCH1 expression during the cell cycle. It is well-known that in G_2_, PLK1 is activated to promote entry into mitosis (see Ref. 14 and references therein). Thus, we sought to find the physiological conditions required to degrade NOTCH1 in the cell cycle context. To this purpose, we conducted synchronization experiments in HaCaT and SCCO22 human cells. A hydroxyurea block and release was performed to synchronize the cells in G_1_/S, and the cell cycle profile was monitored. After the cells were released from the hydroxyurea-induced G_1_/S block, the cells were harvested and subjected to a Western blot analysis. The phosphorylation of Thr-210 was observed strongly at the G_2_ phase of the cell cycle, a pattern inversely correlated with the NOTCH1 expression (Fig. 3, *A* and *B*). However, the inhibition of PLK1 by BI2536 induced the accumulation of NOTCH1 protein (Fig. 3*C*), confirming that PLK1 promotes NOTCH1 down-modulation during the cell cycle. Our data indicate that PLK1 phosphorylates and consequently destabilizes NOTCH1 in the G_2_-M transition. However, to be transformed, in cells under genotoxic stress, the checkpoint response should be down-regulated to tolerate the cellular DNA damage stresses. PLK1 activation regulates the checkpoint activation and allows cells to grow under genotoxic stress (22). Moreover, PLK1 is also known to be involved in promoting resistance to chemotherapeutic regimens with drugs such as doxorubicin (a DNA-intercalating compound) (23). We found that under arsenite treatment, NOTCH1 is continuously degraded, and in this condition, PLK1 is active (Figs. 1 and 2). Notably, a G_2_ phase–specific inactivation of PLK1 after DNA damage has been described. The reason for this inactivation is to promote cell cycle exit to avoid proliferation and entry in mitosis in the presence of damaged DNA. Thus, we investigated whether PLK1 targets NOTCH1 during G_2_ in response to DNA damage. To this end, both HaCaT and SCCO22 cells were synchronized at G_1_/S and then allowed to progress through the cell cycle. At 7 h after the release from G_1_/S (when cells were in G_2_), cells were pulsed with doxorubicin for 1 h to induce DNA damage and harvested 18 h after doxorubicin release (Fig. 4*A*; only HaCaT cells are shown). As expected, induction of DNA damage results in decreased levels of PLK1 and activation of ATM (Fig. 4, *B* and *C*). Notably, when PLK1 was dephosphorylated and inactive, the expression of NOTCH1 was restored, indicating that NOTCH1 expression is up-regulated during the G_2_ damage checkpoint (Fig. 4, *B* and *C*). Interestingly, similar results were obtained in FaDu cells, a SCC cell line with mutated p53, and HeLa cells, an adenocarcinoma cell line with WT p53 (Fig. S6), strengthening the argument that NOTCH1 and PLK1 are inversely correlated during DNA damage response.

### Upon DNA damage in G_2_, NOTCH1 protects cells from apoptosis

**Figure 5.**
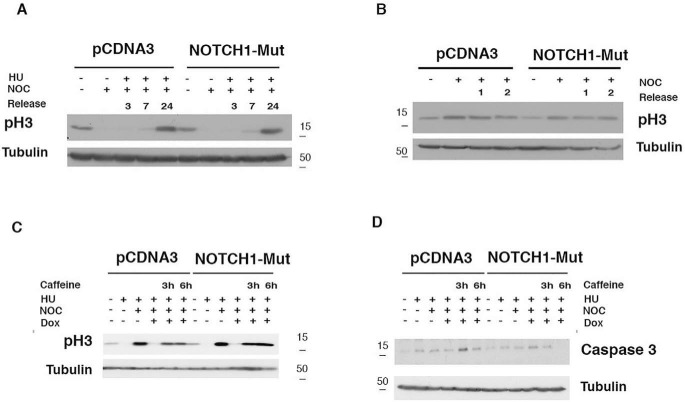
**Overexpression of NOTCH1 mutant unphosphorylable by PLK1 has no effect on cell cycle progression.** *A*, SCCO22 cells were transfected with either control, empty-PCDNA3 vector, or A1791/A2391-NOTCH1-ICD mutant. The cells were synchronized with hydroxyurea (*HU*) for 19 h. At the indicated time points after release, the cells were harvested and subjected to immunoblotting for the indicated proteins. *B*, cells were treated as described for *A*, except that cells were trapped with nocodazole (*NOC*) for 14 h and then released. At the indicated time points after release, the cells were harvested and analyzed with the indicated antibodies. *C* and *D*, SCCO22 cells were transfected with either control, empty-PCDNA3 vector, or A1791/A2391-NOTCH1-ICD mutant. The cells were synchronized with hydroxyurea for 19 h. Cells were released from the hydroxyurea (HU) block and either untreated or treated after 7 h with doxorubicin for 1 h and subsequently grown in the presence of nocodazole and caffeine the last 3 and 6 h. Cells were harvested and subjected to immunoblotting for the indicated proteins.

**Figure 6.**
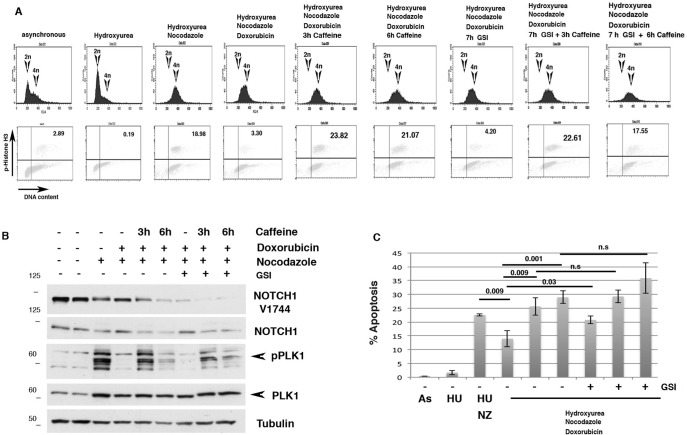
**NOTCH1 expression in recovery from a G_2_ DNA damage arrest.** *A*, HaCaT cells were left untreated or treated with hydroxyurea (*HU*) for 19 h. Alternatively, cells were released after the hydroxyurea block and, 7 h after release, treated with doxorubicin for 1 h and subsequently grown in the presence of nocodazole for 18 h. Following these treatments, caffeine was added for the indicated time periods to allow recovery from the checkpoint-induced arrest 3 and 6 h before harvesting the cells. DNA content and phosphohistone H3 positivity were determined. *B*, cells were treated as described in *A*, and whole-cell lysate was used for Western blotting with the indicated antibodies (*C*). Cells were treated as described in *A*, and percentage of apoptosis was determined by FACS analysis. *n.s.*, not significant; *As*, As_2_O_3_; *NZ*, nocodazole.

To unravel how PLK1 and NOTCH1 might functionally interact, we investigated whether NOTCH1 had a mitotic role. To this end, we made use of Ser-1791/2349 mutant NOTCH1-IC. SCCO22 cells were transfected with either empty vector or NOTCH1-IC Ser-1791/2349 mutant. Cells were synchronized at the G_1_/S and released into the cell cycle; we did not observe any difference in cell cycle progression as phosphorylated histone H3 (pH3) showed the same kinetic during release (Fig. 5*A*) in both control– and NOTCH1-IC Ser-1791/2349 mutant–treated cells. Furthermore, no mitotic delay was detected in cells examined at either early time (1 and 2 h) or at longer times after nocodazole release (Fig. 5*B* and data not shown). We conclude that in this cellular context, NOTCH1 does not alter the G_2_/M transition. Previous observations established that PLK1 plays a critical role in the G_2_ checkpoint recovery following DNA damage (14, 24), and we found that NOTCH1 expression is up-regulated during the G_2_ damage checkpoint (Fig. 4). Thus, we evaluated whether NOTCH1 expression would alter recovery following DNA damage. To test this, cells were synchronized at the G_1_/S and released into the cell cycle. After 6 h from release, cells were treated with doxorubicin to induce the G_2_ damage checkpoint. Later cells were treated with caffeine to abrogate the G_2_ checkpoint response. As expected, we detected an increase of pH3 in empty vector–treated cells after caffeine addition (Fig. 5*C*). Interestingly, NOTCH1-IC mut expression enhanced pH3 expression (Fig. 5*C*). Treatment of cells with caffeine abrogates the G_2_ checkpoint but also promotes mitotic catastrophe and apoptosis (14). Consistently, we found that in empty vector–treated cells, caffeine treatment induced activation of caspase-3, whose expression levels were reduced in NOTCH-IC mut–treated cells (Fig. 5*D*). Although we observed a differential expression of the cleaved caspase-3, neither empty vector– nor NOTCH1-IC mutant–treated cells showed sign of apoptosis after the caffeine addition (data not shown). The mechanism by which DNA-damaged cells escape from apoptosis during the DNA damage checkpoint is poorly understood. Therefore, we wondered whether the requirement of NOTCH1 during the DNA damage–induced G_2_ checkpoint could be restricted to such an anti-apoptotic signaling. To test this, we designed an experimental set-up to examine whether a cell cycle arrest/restart following a DNA damage–induced G_2_ arrest in HaCaT cells would be dependent on the function of NOTCH1. HaCaT immortalized cells were chosen because in this cellular context, conversely to SCCO22 cells, sustained DNA damage checkpoint promotes apoptosis. Thus, HaCaT cells released from a hydroxyurea block were treated with doxorubicin at 7 h after release, a time at which the great majority of the cells had completed S phase (Fig. 6*A*). Using this approach, we were able to obtain a highly synchronous population of cells arrested at the G_2_ DNA damage checkpoint by doxorubicin (Fig. 6*A*). Subsequently, we mimicked checkpoint silencing by the addition of the checkpoint kinase inhibitor caffeine and allowed the cells to enter mitosis in the presence of nocodazole. Notably, doxorubicin treatment of HaCaT cells resulted in lower mitotic index when compared with control cells (Fig. 6*A*, *bottom panels*, *diagrams 3* and *4*). After 3–6 h of caffeine treatment, a significant fraction of cells entered mitosis as judged from phosphohistone H3 staining (Fig. 6*A*, *bottom panels*). When cells entering in the G_2_ damage–induced checkpoint were examined in more detail, a decrease in pPLK1 level and the appearance of NOTCH1 expression were observed (Fig. 6*B*, *lanes 3* and *4*). When we analyzed cell recovery from DNA damage–induced arrest after doxorubicin treatment, we found that G_2_-arrested cells could be forced to enter mitosis following the addition of caffeine. Interestingly, caffeine treatment increased PLK1 expression, indicating that, as previously shown, PLK1 becomes essential for mitotic entry and recovery from a DNA damage–induced G_2_ arrest (24). Consistent with a role for PLK1 in the control of NOTCH1 expression, we found that pPLK1 activation was paralleled by NOTCH1 down-modulation when caffeine was added to induce recovery from a DNA damage–induced G_2_ arrest (Fig. 6*B*). Notably, NOTCH1 does not seem to be instrumental for achieving a DNA damage–induced arrest, because GSI-treated cells efficiently arrested in response to DNA damage (Fig. 6A, *seventh diagram*). Strikingly, when we examined the fate of the damaged cells that are in the DNA damage–induced G_2_ arrest or induced to enter mitosis by the addition of caffeine in the presence of GSI, we found that cell viability was severely affected (Fig. 6*C*). These results demonstrate that NOTCH1 protects cells from DNA damage–induced arrest and that PLK1-mediated degradation of NOTCH1 may be essential for recovery from a DNA damage-induced arrest.

### NOTCH1 promotes inflammatory cytokine secretion in cancer cells that undergo growth arrest in response to DNA damage

**Figure 7.**
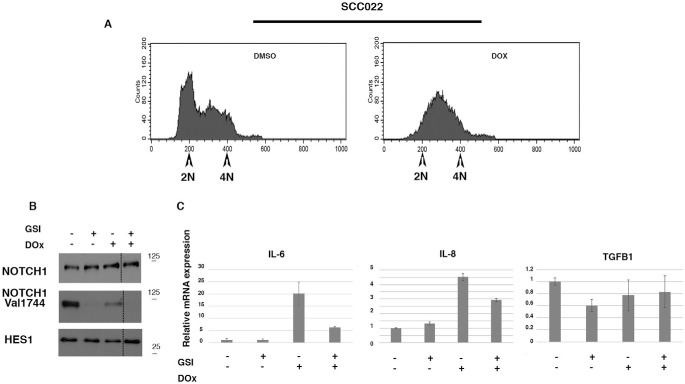
**NOTCH1-dependent increased expression of IL-6 and IL-8 during DNA damage–induced growth arrest.** SCCO22 cells were treated with doxorubicin (*DOX*), following which either DMSO or GSI was added, and cells were maintained in culture for a further 24 h. In *A*, cells were analyzed by FACS analysis. *B*, cells were treated as described in *A*, and whole-cell lysate was used for Western blotting with the indicated antibodies. Additional samples present on the gel were cropped as indicated by *dashed lines*. In *C*, cells were treated as described for *A*, and total RNA was used for quantitative RT-PCR with the indicated probe.

Induction of cell cycle arrest in response to DNA damage represents a protective mechanism against harmful mutations but also promotes apoptosis (14, 24). We found that NOTCH signaling protects immortalized HaCaT cells from DNA damage–induced apoptosis. Conversely, we observed that in the squamous cell carcinoma cell line, SCCO22, induction of DNA damage by doxorubicin treatment promotes a permanent cell cycle arrest with no sign of apoptosis (Fig. 7 and data not shown). In response to DNA damage, growth-arrested cancer cells also develop a secretory phenotype that alters tissue microenvironments and might stimulate tumor growth *in vivo* (25). Among the secreted factors, IL-6 and IL-8 are of particular interest. These cytokines have been shown to promote tumorigenesis by regulating processes associated with tumorigenesis raging from cancer metabolism to metastasis (25, 26). Therefore, we wondered whether NOTCH1 during DNA damage–induced G_2_ checkpoint could be involved in such secretory signaling. To test this, SCCO22 cells were treated with doxorubicin to induce the G_2_ damage checkpoint (Fig. 7*A*). Later, cells were treated with GSI to inhibit NOCTH signaling (Fig. 7, *B* and *C*). As expected, we detected an increase of IL-6 and IL-8 in doxorubicin-treated cells (Fig. 7*C*). Interestingly, GSI treatment decreased both IL-6 and IL-8 expression (Fig. 7*C*) but not TGFB1 that has been associated with the development of a secretory phenotype of cancer cells. Thus, these data support a model in which the epithelia cancer cells, SCCO22, use Notch signaling to support a secretory phenotype.

## Discussion

*NOTCH1* activity plays pivotal roles in signaling for diverse cellular process, such as cell differentiation, stem cell renewal, proliferation, and transformation (1, 8, 27). *NOTCH1* signaling has been reported to have a contradictory role in cell transformation (4, 8). However, a widely accepted model implies that the impact of *NOTCH1* signaling is highly context-dependent, and it can have opposite effects in different systems. We have used arsenite-induced malignant transformation of a human epithelial cell line as an *in vitro* model to study the mechanisms that can result in NOTCH1 role and function alterations (12). We previously demonstrated that whereas arsenite-mediated apoptosis of immortalized keratinocytes was associated with NOTCH1 down-regulation, arsenite-mediated transformation of these cells was characterized by increased NOTCH1 stability (12). We found that *NOTCH1* regulates cellular metabolism and apoptosis, which in turn differentially impact cell proliferation and cell transformation (12). Consequently, the cellular genetics/context may impinge on the antagonistic duality of *NOTCH1* function. We presented evidence indicating that *FBXW7* is required for the differential expression of NOTCH1 during arsenite-mediated transformation, indicating that kinases and biochemical pathways could be involved in NOTCH1 phosphorylation in tumors. Given that NOTCH1 stability and signaling are controlled by its phosphorylation (21), the study of kinases that could be implicated in this post-translational modification could help to elucidate the mechanisms controlling NOTCH1 dichotomy in cancer development. In this study, the effects of 378 cellular kinase inhibitors on NOTCH1 transcriptional activity and protein stability after arsenite treatment were investigated. Our findings indicate that multiple kinases implicated in various cell signaling pathways can participate in these outcomes: FAK, IKKB, PKA, ATM, ATR, SRC, p38, m-TOR, GSK1, c-MET, CDK1, ALK, PLK1, AURKA/B, CSF1R VEGFR, and JAK. To understand how the identified kinases might have an impact on NOTCH1, we performed a network analysis to investigate all possible direct and indirect interactions among them. The central component of the shortest path network was the protein PLK1, which is a central regulator of cell division required for several events of mitosis and cytokinesis (13, 14). Whereas in nondamaged cells, the PLK1 pathway is involved in G_2_/M transition, PLK1 was shown to be a direct target of the G_2_ DNA damage checkpoint. Indeed, in response to a wide range of DNA-damaging agents, PLK1 was shown to be catalytically inactivated. Moreover, this inhibition was shown to depend on functional ATM or ATR (14). Such control of the cell cycle machinery may be critically important to prevent a premature restart of the cell cycle following genotoxic stress. However, in addition to being a target of the DNA damage checkpoint, PLK1 was also shown to regulate cell cycle progression after a damage-induced cell cycle arrest. In this context, cells escape the DNA damage checkpoint arrest in a process called “adaptation.” Such a mechanism allows damaged cells to eventually divide and possibly survive and undergo transformation (14, 15). Consistent with the above observation, we found that when challenged with arsenite, cells were G_2_-arrested. The data presented here show that NOTCH1 is a novel substrate of PLK1. Additionally, we found that in an unperturbed cell cycle, PLK1 appears to be involved in NOTCH1 down-modulation at the mitotic entry. Interestingly, we observed an increase in the levels of Thr-210 PLK1 expression, which indicates that PLK1, by facilitating tolerance to arsenite-induced genotoxic stress, might favor arsenite-induce cell transformation. Notably, the coordination of this pathway becomes critical for both DNA damage checkpoint and mitotic entry in cells recovering from a DNA damage–induced arrest (28). Although its exact involvement remains to be established, in arsenite-induced transformation, NOTCH1 represents a checkpoint mediator targeted by PLK1 to silence the DNA damage checkpoint in a condition in which damage persists for long periods of time. Thus, PLK1 activation initiates an escape program from checkpoint-mediated arrest prior to completion of damage repair. NOTCH1 inactivation is part of the PLK1-associated adaptation program to DNA damage that can result in enhanced cell death (*e.g.* through mitotic catastrophe) but at the same time may allow the propagation of defects in the genome to the daughter cells that may contribute to cell transformation. Although our observations necessitate further analysis to understand how deregulation of the NOTCH1 pathway impacts signaling that responds to DNA damage, we provide evidence that Notch signaling is altered but not abolished in SCC cells. We found that NOTCH signaling might contribute to the secretory phenotype of epithelial cancer cells. Thus, the dual role of Notch in cancer biology is undoubtedly complex and tumor type–independent. It is important to recognize that even in a single type of tumor, there is plasticity in Notch function that deserves greater attention.

## Experimental procedures

### Cell culture and transfection

HaCaT-S immortalized and HaCaT-R cells were described previously (12). Culture cells 70–80% confluent were maintained in modified low-calcium medium and transfected using the Lipofectamine transfection reagent (L-006119-00, Thermo Scientific/Dharmacon (Lafayette, CO)) according to the manufacturer's instructions (Thermo Fisher Scientific). Cells were analyzed at the indicated times after transfection by either RT-PCR analysis or Western blotting, as indicated (12, 29). SCCO22 were kindly provided by Dr. Caterina Missero (Università degli Studi di Napoli, Naples, Italy). HeLA and FaDu were kindly provided by Dr. Angelo Peschiaroli (CNR, Rome, Italy).

### Reagents and immunoblotting

The following reagents were purchased from Santa Cruz Biotechnology: Fbxw7 and tubulin. In addition, we used Notch1 Val-1744, Notch1 D1E11, PLK1 208G4, and PLK1(Thr-210) from Cell Signaling Technology (Beverly, MA). γ-Secretase inhibitor IX (DAPT), was purchased from Calbiochem (Merck KGaA), dissolved in DMSO, and stored at −20 ºC until use. All cell extracts were prepared as described previously (30) and according to the manufacturer's instructions for detection of phospho-ERK (Cell Signaling Technology). The kinase library of 378 structurally diverse, cell-permeable kinase inhibitors was purchased from Selleckchem (Houston, TX) (catalogue no. L1200) (Table S1).

Notch1-ICD encodes the expression of human Notch1-IC from amino acid 1757 to 2555 and has been described previously (9). GST-NOTCH1-IC plasmid encodes the GST-Notch1-IC fusion protein encoding the mouse NOTCH1-IC region 1753–2531 was kindly provided by Dr. Lendhal (Karolinska Institute, Stockholm, Sweden) and described previously (31). The plasmids containing mutations in Notch1-ICD encoding the expression of human Notch1-IC from amino acid 1757 to 2555 were generated using the QuikChange II XL site-directed mutagenesis kit (Thermo Fisher Scientific) and verified by sequencing.

### Kinase library screening

Transient transfection/promoter activity assays were performed using a Dual-Luciferase/*Renilla* reporter assay system (Promega). All conditions were tested in triplicate samples, and a 12xCSL-luciferase reporter vector responsive to *NOTCH* signaling was co-transfected with either pcDNA3 as control or NOTCH1-IC vector. At 24 h after transfection, cells were treated with compounds in triplicate at 10 μm, and a luciferase assay was conducted in the presence of 5 μm arsenite. The results were normalized against *Renilla* luciferase. To control for cytotoxic effect of the compounds, when the *Renilla* luciferase activity was reduced to <25% of the activity seen with the vehicle-treated controls and the survival rate was less than 75%, those compounds were excluded from further analysis. Those compounds showing a ≥50% recovery of luciferase activity were further tested in increasing amounts. In this second step, each compound was tested in increasing amounts (1, 5, and 10 μm) in the presence of 5 μm arsenite. All compounds were further tested for their ability to rescue NOTCH1 expression after arsenite treatment by Western blotting at 10 μm in the presence/absence of 5 μm arsenite.

### PLK1 kinase assay

For the PLK1 kinase assay, GST-Notch1 fusion protein was expressed in *Escherichia coli* BL21 strain and purified using standard procedures. PLK1 kinase assays were carried out using the PLK1 activity assay reagent kit purchased form SignalChem (Richmond, Canada) according to the manufacturer's instructions.

### Cell cycle analysis

To analyze mitotic entry, cells were fixed and stained with propidium iodide and an antibody against phosphohistone H3 (Ser-10) using the FlowCellect^TM^ cell cycle kit for G_2_/M analysis (EMD Millipore, Darmstadt, Germany). The percentages of M-phase cells and cellular DNA content were determined by flow cytometry using a FACSCalibur flow cytometer (BD Biosciences).

### Synchronization and recovery from DNA damage

HaCaT, SCCO22, FaDu, and HeLa cells were grown in Dulbecco's modified Eagle's medium and RPMI supplemented with 10% fetal calf serum, 100 units/ml penicillin, and 100 μg/ml streptomycin. For the synchronization experiments, cells were incubated in hydroxyurea (1.5 mm) for 19 h to arrest cells at the G_1_/S transition. Where indicated, the G_2_/M DNA damage checkpoint was activated by treating cells with 0.5 μm doxorubicin for 1 h at 7 h after release from a hydroxyurea block. Doxorubicin was washed away thoroughly, and immediately after washing, nocodazole (250 ng/ml) was added to the culture medium. 18 h after washing away doxorubicin, all cells were arrested in G_2_ as judged from FACS analysis. To inactivate DNA damage signaling and allow mitotic entry, caffeine (5 mm) was added to inhibit ATR and ATM checkpoint kinases. The continuous presence of nocodazole prevented exit from mitosis and allowed accumulation of cells in mitosis. To inactivate NOTCH1 signaling, GSI (5 μm) was added 30 min before doxorubicin treatment and then maintained until cells were harvested for further analysis.

## Author contributions

C. D. B. and C. T. formal analysis; C. D. B., A. Z., M. F., G. M., S. Cialfi, N. V., D. Benelli, and C. T. investigation; C. D. B., A. Z., and C. T. methodology; S. Checquolo, D. Bellavia, R. P., and I. S. resources; D. Benelli and C. T. conceptualization; C. T. data curation; C. T. funding acquisition; C. T. writing-original draft; C. T. project administration; C. T. writing-review and editing.

## Supplementary Material

Supporting Information

## Footnotes

4 The abbreviations used are:

PS: presenilin
ICD: intracellular domain
HNSCC: head and neck squamous cell carcinoma
PLK1: Polo-like kinase 1
GSI: γ-secretase inhibitor
pH3: phosphorylated histone H3
mut: mutant
ERK: extracellular signal–regulated kinase.
