# Supplementary material for: PLK1 targets NOTCH1 during DNA damage and mitotic progression
Source: J Biol Chem. 2019 Oct 9;294(47):17941–50. doi: 10.1074/jbc.RA119.009881 (PMC6879332; doi:10.1074/jbc.RA119.009881)
Supplement: Supporting Information [file supp_294_47_17941__index.html]

PLK1 targets NOTCH1 during DNA damage and mitotic progression. — Molecular mechanism of Notch1 duality — PLK1 targets NOTCH1 during DNA damage and mitotic progression — Molecular mechanism of Notch1 duality — Supporting Information 

# PLK1 targets NOTCH1 during DNA damage and mitotic progression

## Supporting Information

- Supporting Information (to be published online) - Supplemental figures
- Supplemental Table - Kinase library descirption
