# Supplementary material for: PLK1 targets NOTCH1 during DNA damage and mitotic progression
Source: J Biol Chem. 2019 Oct 9;294(47):17941–50. doi: 10.1074/jbc.RA119.009881 (PMC6879332; doi:10.1074/jbc.RA119.009881)
Supplement: Supporting Information [file supp_RA119.009881_153654_1_supp_400887_pydpbw.docx]

**Supporting Information**

**Title:**

# PLK1 targets NOTCH1 during DNA damage and mitotic progression.

#

Authors

Carlo De Blasio^1#^, Azzurra Zonfrilli^1^, Matteo Franchitto^1^, Germano Mariano^1^, Samantha Cialfi^1^, Nagendra Verma^1^, Saula Checquolo^2^, Diana Bellavia^1^, Rocco Palermo^1^, Dario Benelli^1^, Isabella Screpanti^1^ and Claudio Talora^1^*

^1^Department of Molecular Medicine; Sapienza University of Rome; Rome, Italy; Rome, Italy; ^2^Department of Medico-Surgical Sciences and Biotechnology, Sapienza University, Latina, Italy;

Contact Information: C. Talora, Sapienza University of Rome, Viale Regina Elena 291 00161 Rome ITALY E-mail: [claudio.talora@uniroma1.it](mailto:claudio.talora@uniroma1.it)

S1


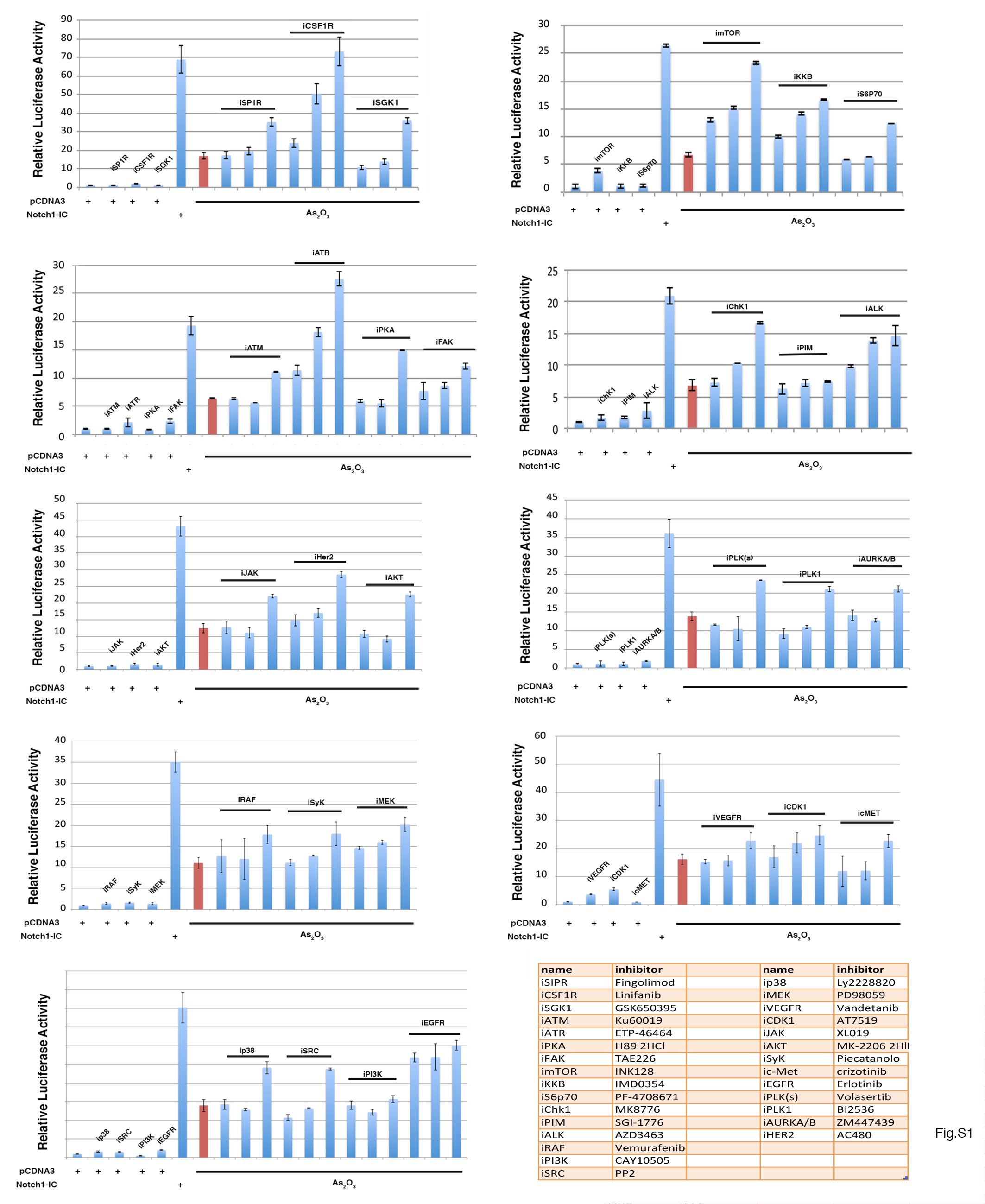


**Figure S1.** A) HaCaT cells were co-transfected with the *NOTCH* responsive promoter 12XCLS and the NOTCH1-IC plasmid plus 5μM As_2_O_3_ and increasing amounts of the indicated inhibitors. 1, 5 10 μM. As control cells were co-transfected with the *NOTCH* responsive promoter 12XCLS and the pDNA3 plasmid plus 5μM of the indicated inhibitors.

S2


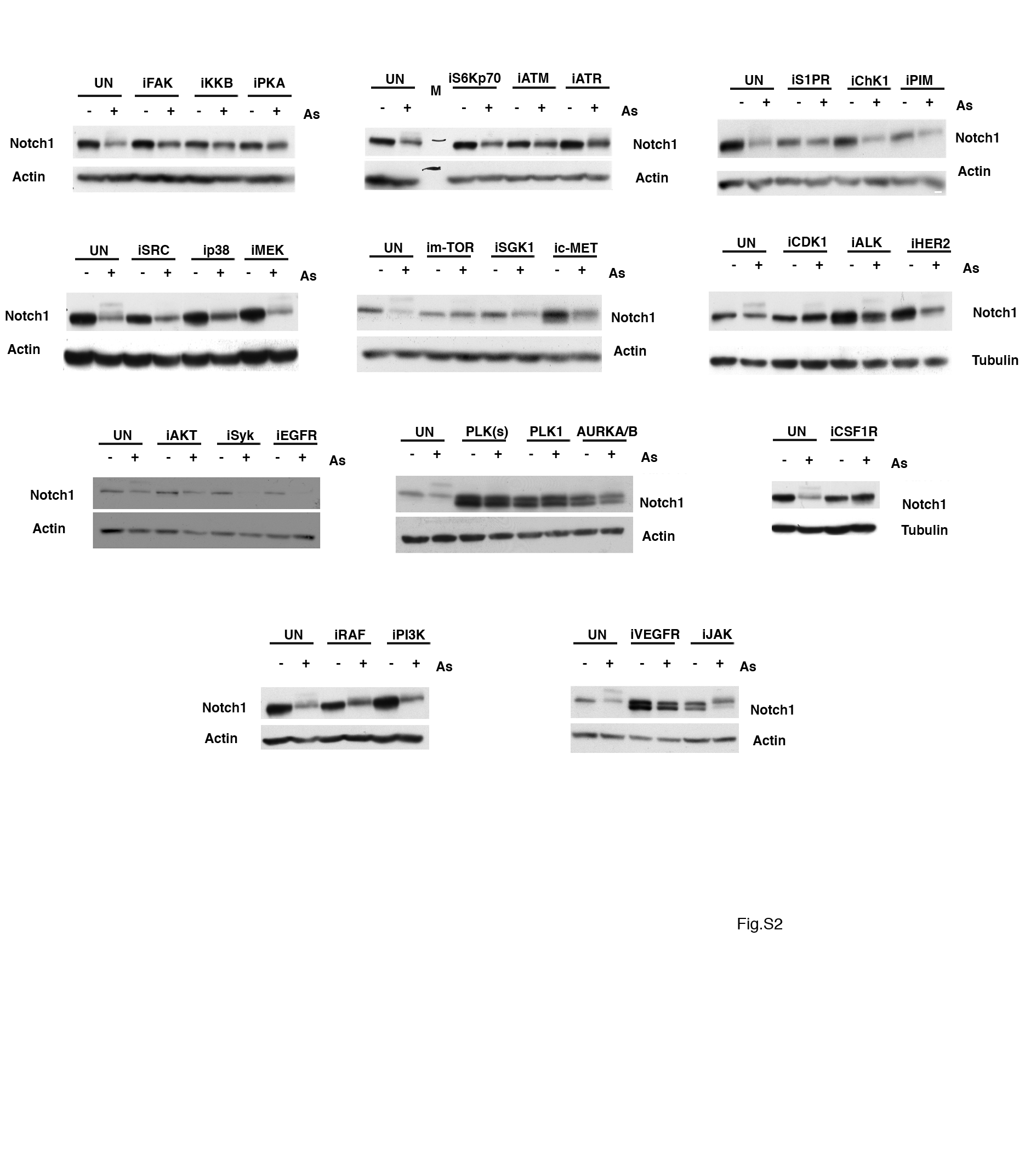


**Figure S2**. A) HaCaT cells were treated with either DMSO or 5μM As_2_O_3_ alone or in combination with the indicated inhibitors (10μM). Cell extracts were analyzed by western blot.

S3


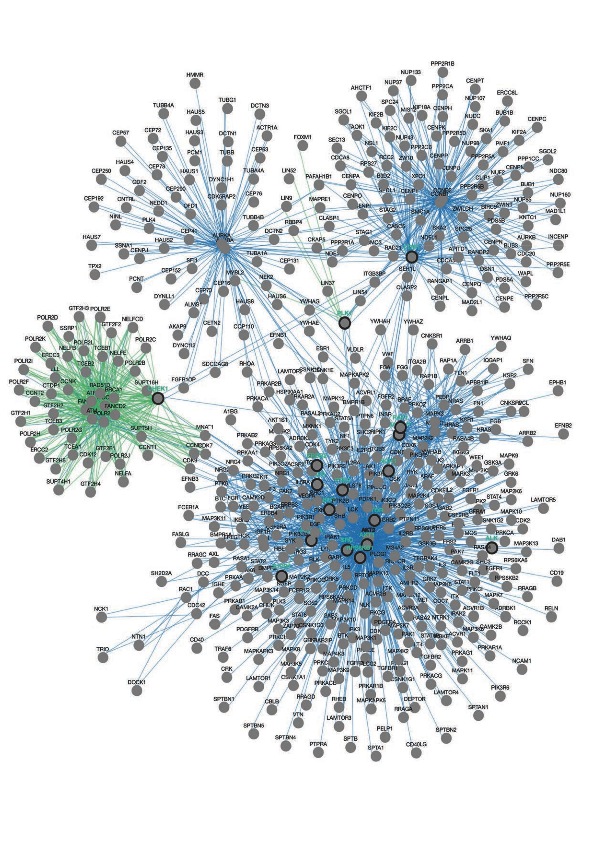


**Figure S3.** Pathway commons network visualizer (PCViz) (<http://www.pathwaycommons.org>) was used to detect functional interaction among the kinases identified in the screening experiments.

S4


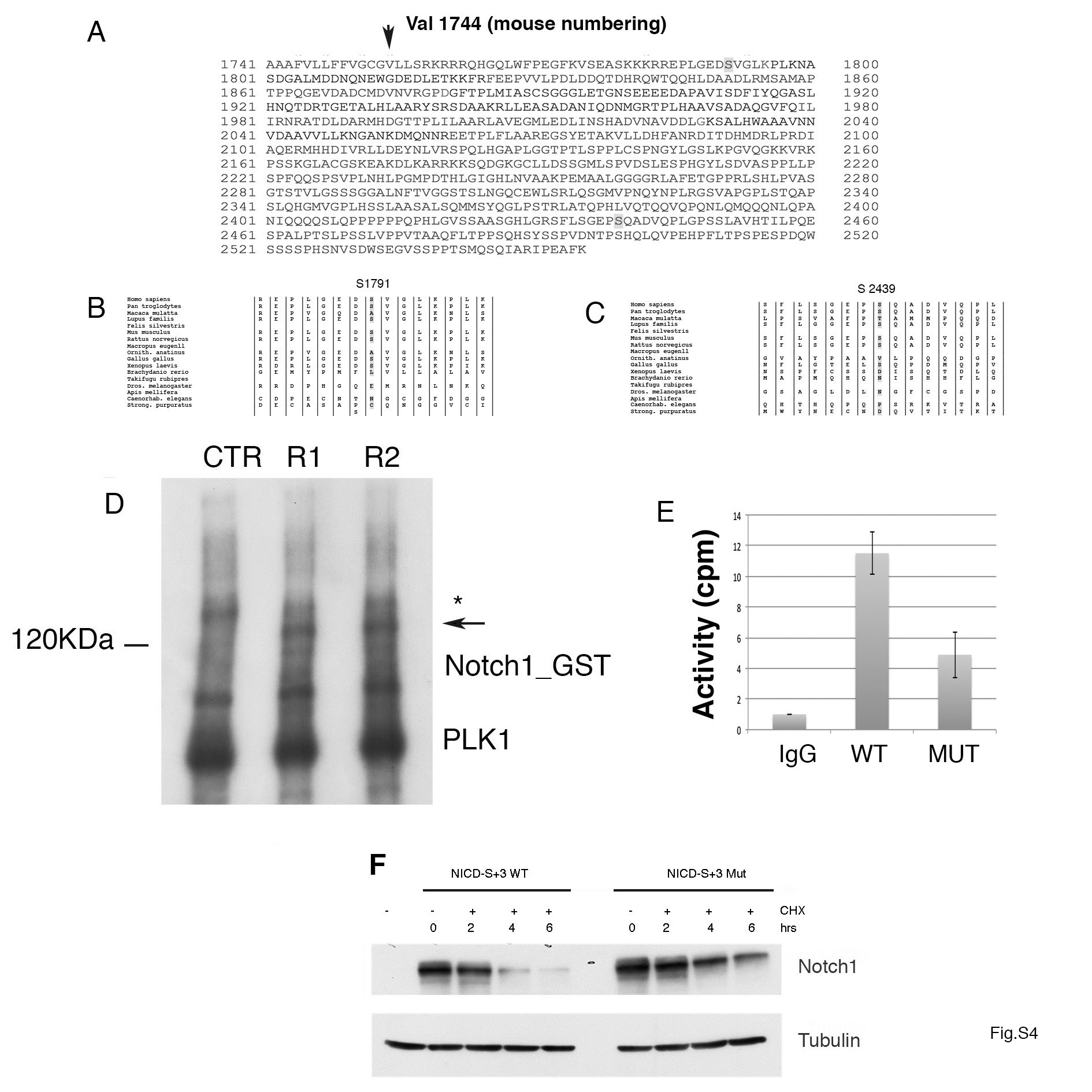


**Figure S4. Direct In Vitro phosphorylation of Notch1 by PLK1.**

**A**) Phosphorylation sites identified in NOTCH1-IC using KINEXUS and GPS-Polo 1.0 platform. (**B-C**) Evolutionary conservation of Ser1791 and Ser 2439 in the Notch1-IC sequence, analysis was performed by Kinexus platform (http://www.phosphonet.ca) (**D**) GST-NOTCH1-IC 1754-2555 was incubated with or without PLK1 followed by in vitro kinase assays. Fractions of the same reactions were analyzed by 7.5% SDS-PAGE. Experiment was performed in two replicates R1 and R2. **E)** NOTCH1-ICD was immunoprecipitated from HaCaT cells transfected with either WT and NOTCH1-IC mutant protein and analyzed by *in vitro* kinase assays as indicated in D except that the assay reaction was analysed by spotting the reaction mixture onto strips of P81 paper and analyzed in a scintillation counter. **F)** HaCaT cells were transfected with plasmids expressing WT-NOTCH1-ICD and mutant A1791/A2391-NOTCH1-ICD for 24 h, then treated with 100μm Cycloheximide for the indicated periods of time. The levels of NOTCH1 in the cell lysates were determined by Western blotting.

S5


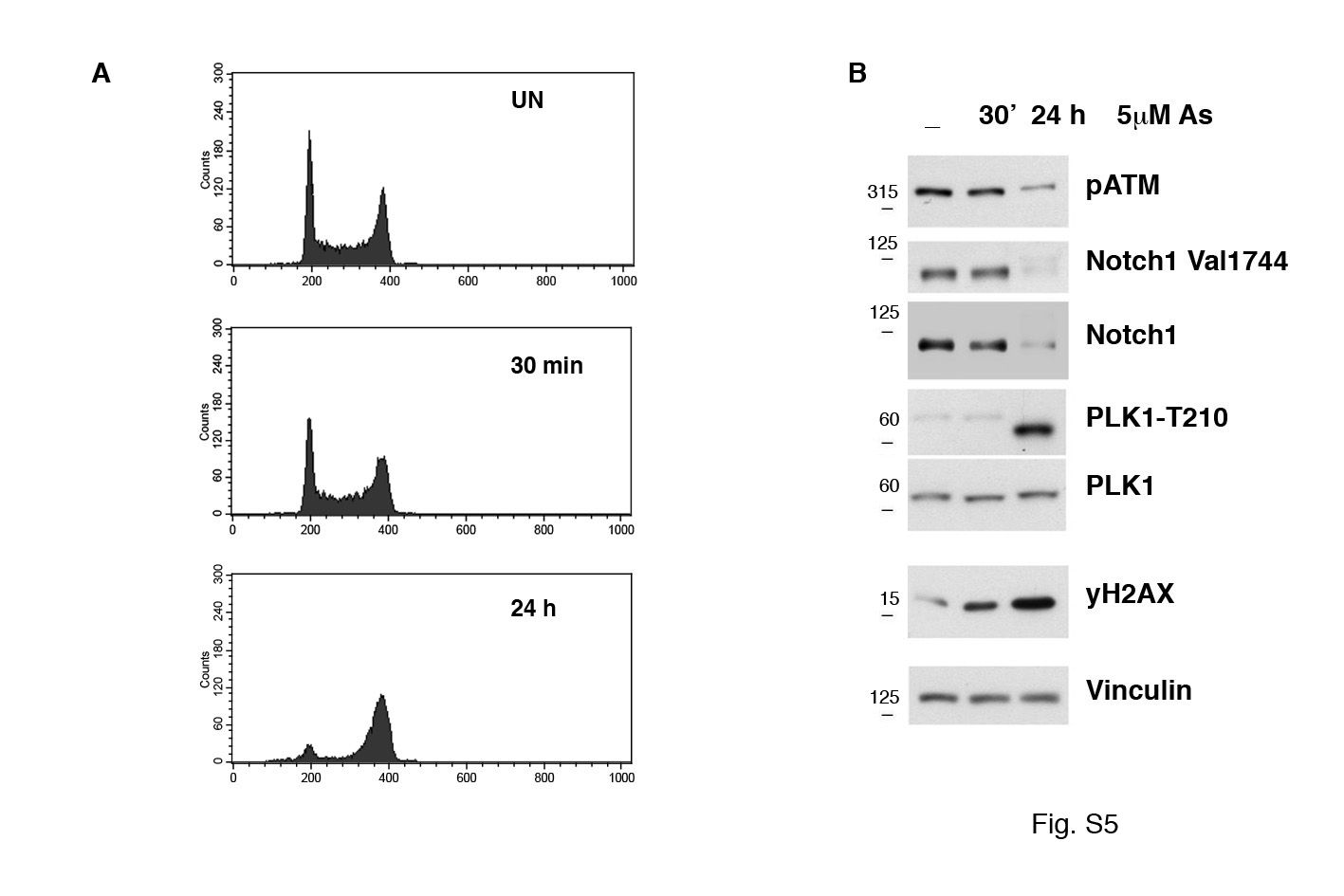


**Figure S5.** **PLK1 and NOTCH1 expression are inversely correlated in in As_2_O_3_-treated cells**. **A)** HaCaT cells were treated for 30 min or 24 hr with Arsenite, then cells were collected and cell cycle analyzed by FACS. **B)** HaCaT cells were treated with As_2_O_3_ for 30 min or 24 hrs and analyzed by immunoblot with the indicated antibodies.

S6


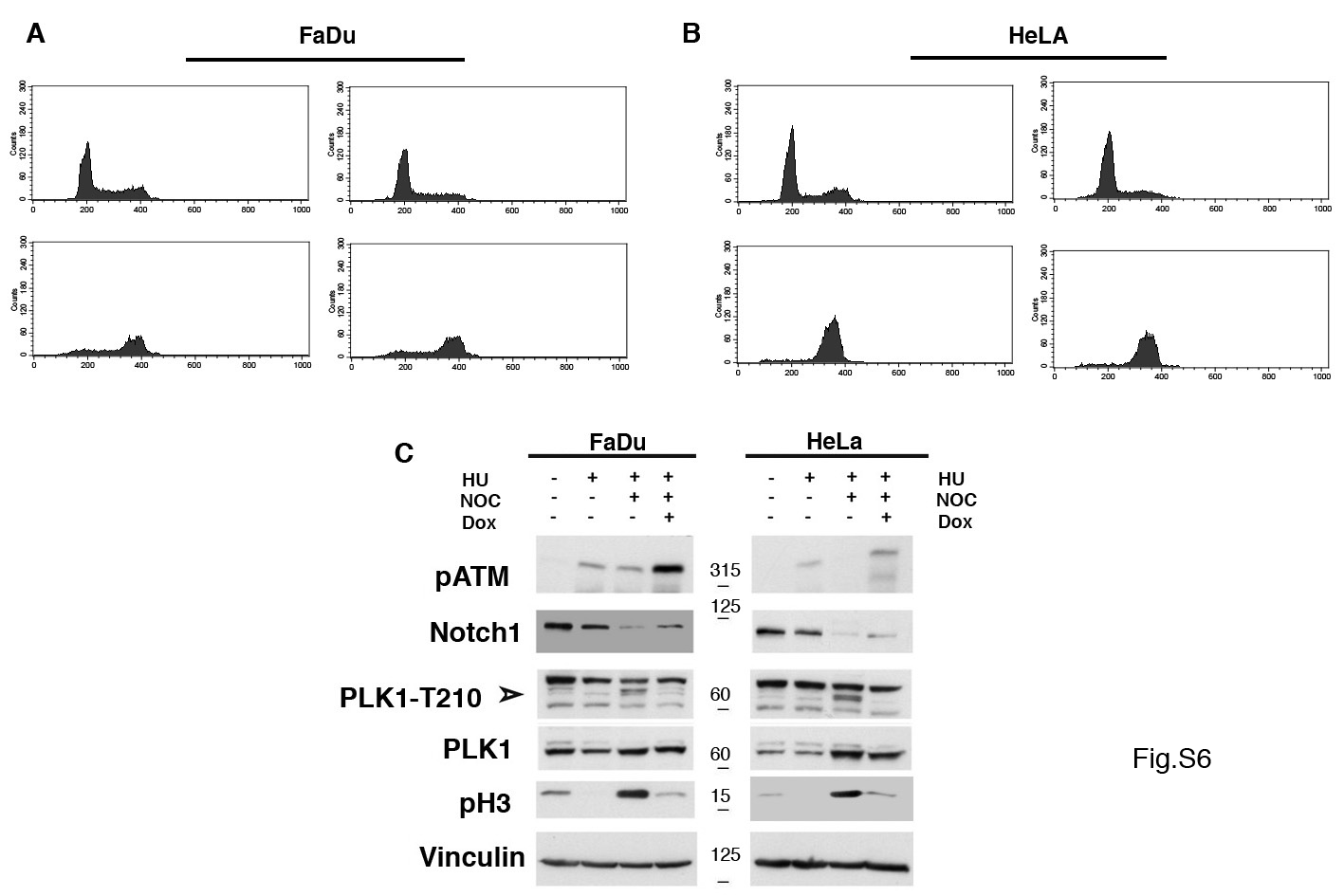


**Figure S6.** **NOTCH1 expression in G2 DNA Damage Arrest**. **A,B,)** FaDu and HeLa cells were left untreated (diagram 1) or treated with Hydroxyurea for 19 hrs (diagram 2). Alternatively, cells were released from the HU block and either untreated or treated after 7 hrs with Doxorubicin for 1 hr and subsequently grown in the presence of Nocodazole for 18 hrs. Following these treatments, cells were collected at the indicated time-points after release from G1/S, cell cycle analyzed by FACS. **C)** FaDu and HeLa cells were treated as described in panel A, B and analyzed by western blot with antibodies against the indicated proteins. Shown are the representative results from at least 3 independent experiments.
